# Environmental Biofilms as Reservoirs for Antimicrobial Resistance

**DOI:** 10.3389/fmicb.2021.766242

**Published:** 2021-12-13

**Authors:** Gabriela Flores-Vargas, Jordyn Bergsveinson, John R. Lawrence, Darren R. Korber

**Affiliations:** ^1^Food and Bioproduct Sciences, University of Saskatchewan, Saskatoon, SK, Canada; ^2^Environment and Climate Change Canada, Saskatoon, SK, Canada

**Keywords:** biofilms, antibiotic resistance, reservoirs, environmental resistome, bacteriophage, sub-minimum inhibitory concentration

## Abstract

Characterizing the response of microbial communities to a range of antibiotic concentrations is one of the strategies used to understand the impact of antibiotic resistance. Many studies have described the occurrence and prevalence of antibiotic resistance in microbial communities from reservoirs such as hospitals, sewage, and farm feedlots, where bacteria are often exposed to high and/or constant concentrations of antibiotics. Outside of these sources, antibiotics generally occur at lower, sub-minimum inhibitory concentrations (sub-MICs). The constant exposure to low concentrations of antibiotics may serve as a chemical “cue” that drives development of antibiotic resistance. Low concentrations of antibiotics have not yet been broadly described in reservoirs outside of the aforementioned environments, nor is the transfer and dissemination of antibiotic resistant bacteria and genes within natural microbial communities fully understood. This review will thus focus on low antibiotic-concentration environmental reservoirs and mechanisms that are important in the dissemination of antibiotic resistance to help identify key knowledge gaps concerning the environmental resistome.

## Introduction

Antibiotic resistance is a well-recognized major threat to global public health. Many studies have surveyed antibiotic resistant bacterial strains or their corresponding genes in hotspots or reservoirs of antimicrobial resistance such as hospitals, waste-water treatment plants (WWTP), sewage systems, and animal feeding operations, where antibiotics are commonly found at high concentrations (Bengtsson-Palme et al., 2018; Burcham et al., 2019; Kraemer et al., 2019; Maestre-Carballa et al., 2019; Xiang et al., 2020). In particular, attempts have been made to correlate the concentration of antibiotics with the occurrence of antibiotic resistant bacterial strains or genes in these hotspots (Rodriguez-Mozaz et al., 2015; Pärnänen et al., 2019).

The complete set of resistance-related genes in a particular system is termed the environmental resistome, and the determination of its risk has been proposed by Martínez et al. (2015), where antibiotic resistance genes (ARGs) are ranked depending on the associated public health risks they might pose (Bürgmann et al., 2018; Asante and Osei Sekyere, 2019). It is known that constant exposure of microbial communities to higher concentrations of antibiotics exerts a selective pressure on the environmental resistome (Ebmeyer et al., 2021), and thus it is also expected that constant exposure to “low-level” or sub-inhibitory concentrations of antibiotics may similarly play important roles in driving antibiotic resistance in environmental microbial communities such as freshwater biofilms and agriculture-impacted environments (Rodriguez-Mozaz et al., 2015; Abe et al., 2020).

Agricultural practices such as irrigation, soil fertilization and animal production are major sources of antibiotic resistance transmission to soil and water resistomes (Manaia et al., 2020) since intensive agriculture operations are more likely to carry ARGs, antibiotic-resistant bacteria (ARB) and run-off chemicals. Even though antibiotics from agriculture run-off can be found at sub-inhibitory concentrations in receiving water bodies or downstream systems, constant exposure over long time periods will contribute to selective pressure for antibiotic resistance in environmental microbial communities such as freshwater biofilms (Andersson and Hughes, 2012; Manaia et al., 2020; Sasikaladevi et al., 2020).

One often overlooked dimension of biofilms is their virome, or the complete assemblage of viral particles that exist within a particular biofilm consortium. The ecological roles of viruses in the spread of antibiotic resistance are not yet fully understood (Bekliz et al., 2019; Palermo et al., 2019). Though it is known that bacteriophage can spread antibiotic resistance or virulence genes *via* transduction, the role of bacterial viruses as potential vectors for environmental antibiotic resistance dissemination is still an ongoing debate (Parsley et al., 2010; Enault et al., 2017; Petrovich et al., 2020). Overall, more studies are required to resolve the complexity of environmental resistomes in pursuit of minimizing the spread of antibiotic resistance (Quinlan et al., 2011; Kraemer et al., 2019; White and Hughes, 2019).

## River Biofilms as Environmental Reservoirs of Antimicrobial Resistance

### Characteristics and Components of Biofilms

Biofilms are ubiquitous throughout nature and may be found attached to any submerged surface or solid-liquid interface, such as stones, wood, shells, sediment, or on other living aquatic organisms (Lawrence et al., 2012, 2015). Occurring at the base of aquatic food chains, biofilms represent a key microbial compartment that serves vital roles for ecosystem functioning due to their biomass and diversity. The biofilm’s multiple trophic levels are formed by primary producers and heterotrophic prokaryotes and eukaryotes which commonly include bacteria, algae, archaea, fungi and Protista (Villanueva et al., 2011; Proia et al., 2013; Lawrence et al., 2015).

The structure of biofilms is highly variable and influenced by myriad factors, but typically characterized by microcolonies of bacterial cells encased in an extracellular polymeric substance (EPS) matrix and separated from other microcolonies by interstitial voids (water channels) (Caldwell et al., 1992, 1993; Lewandowski, 2000; Donlan et al., 2002). Besides polysaccharides, the biofilm EPS matrix also includes proteins, lipids, extracellular DNA and even inorganic solids (Flemming and Wingender, 2010). It can account for 50–90% of the total organic carbon of biofilms and some consider it to be the primary matrix material of the biofilm (Flemming and Wingender, 2010; Fulaz et al., 2019).

### Biofilm Formation

The biofilm “lifestyle” offers multiple potential benefits to member microorganisms, including enhanced nutrient uptake, limited diffusion of potentially deleterious compounds by the EPS matrix, stable positioning in optimal locations, beneficial interactions between complementary neighbors, and creation of chemically stable microniches (Lawrence et al., 2012, 2015; Orazi and O’Toole, 2019). The fast-growing and sessile nature of the microbial communities, as well as their dependence on environmental physical and chemical conditions, make them ideally suited as indicators of disturbances in river systems, from tracking increases in temperature to the presence of inorganic pollutant chemicals and materials from the surrounding environment (Romaní, 2009; Ramanan et al., 2016; Tu et al., 2020). Because of their autotrophic and heterotrophic metabolism, biofilm communities are major sites for the uptake, storage and transformation of fluvially-dissolved organic matter and nutrients, thus playing a major functional role in stream ecosystems (Romaní et al., 2004; Bott et al., 2006).

In addition to the EPS matrix providing structural rigidity and protection, environmental factors such as water flow velocity, availability and types of nutrients, temperature, suspended solids, and light availability contribute to shaping the three-dimensional structure (often termed “architecture”) of biofilm microcolonies and the abundance and nature of inhabiting bacteria (Lawrence et al., 1991, 2004, 2015; Mangalappalli-Illathu et al., 2008a; Wingender and Flemming, 2011; Tu et al., 2020), as well as the extent and thickness of biofilm development. The development of the biofilm may further be influenced by the growth of algae, cyanobacteria, protozoa and the EPS matrix, particularly the latter, as it has been shown to strongly impact the development of the biofilm’s microenvironmental conditions (Romaní et al., 2004, 2014; Flemming and Wingender, 2010).

### Horizontal Gene Transfer

Bacterial cells within biofilm communities have the capacity to respond, when challenged with stressors like antibiotic discharge (Schroeder et al., 2017), using various mechanisms. Importantly, pathogenic virulence factors and antibiotic or stress resistance factors share features that are involved in efflux pumps, porins, cell-wall alterations and two-component sensory systems that activate or repress the expression of genes or gene operons (Beceiro et al., 2013; Schroeder et al., 2017). ARGs are often co-located with pathogen virulence factors in biofilm-producing organisms, thereby aiding in the selection for, and spread of, antibiotic resistance in pathogenic organisms (Schroeder et al., 2017) and often leading to multiple drug resistance (MDR).

ARGs, which comprise the “resistome” of a given metagenome dataset (Maestre-Carballa et al., 2019) proliferate *via* horizontal gene transfer (HGT). The “mobilome,” or all gene elements related to HGT, is defined as all detectable horizontal mobile genetic elements (MGE) within a given metagenomic dataset (Slizovskiy et al., 2020) and includes plasmids, transposons, insertion sequences, bacteriophage, integrons and extracellular DNA (e-DNA). MGEs can be transferred between distantly related bacteria, including different phyla, and therefore do not tend to carry genes essential for cell metabolism or survival (Wellington et al., 2013). Recently, Partridge et al. (2018) published an extensive review on MGEs in clinically relevant species and their association with antimicrobial resistance, which we recommend readers to review.

Due to its importance in clinical infections and tendency to form biofilms, *Pseudomonas aeruginosa* has often been used as a model for the study of antibiotic resistance and tolerance in biofilms. Therefore, most of the molecular mechanisms of antibiotic resistance known today are based on *in vitro* studies first benchmarked using *P. aeruginosa* biofilms (Brooun et al., 2000; Hall and Mah, 2017). In addition, Gram-negative bacteria are intrinsically more resistant to antibiotics (i.e., vancomycin) than Gram-positives, due to the relative impermeability of the Gram-negative cells’ outer membrane (Hall and Mah, 2017). Conjugation, presumably the principal route of HGT in sessile bacterial communities, has been documented many times in hospital studies (Lerminiaux and Cameron, 2019; Abe et al., 2020). Higher rates of e-DNA transformation also occurs in biofilms, with this e-DNA being concentrated within the biofilm EPS (Madsen et al., 2012). For example, Lehmann et al. (2016) recorded the prevalence of MGE in freshwater biofilms after sub-minimum inhibitory concentrations (sub-MIC) exposure to treated sewage effluent (2.5 ppm), and determined that biofilms which were exposed to sewage effluent had significantly higher prevalence of Class 1 integrons (Table 1). Transfer of resistance elements may also be carried out *via* transduction by phage, though this mechanism is notably underexplored in biofilm communities.

**TABLE 1:** Examples of biofilms exposed to low antibiotic concentrations.

Antibiotic	Low AB concentration	Description	ARGs	MGEs	Polymicrobial biofilm	References
Trimethoprim	1.2–10.4 ng/g	Exposure to the discharge from WWTPs		Class 1, 2, 3 integrons	Yes	Aubertheau et al., 2017
Sulfamethoxazole	1.3–20.1 ng/g					
Levofloxacin + ofloxacin	10–276 ng/g					
Ofloxacin	421–649 ng/L	Natural biofilms exposed to a pressurized sewage pipe.	qnrS, sul1, sul2, blaTEM, blaKPC, ermB, tetM, tetW	intI1	Yes	Auguet et al., 2017
Ciprofloxacin	2905–1379 ng/L					
Norfloxacin	652–731 ng/L					
Tobramycin	1 mg/ml; 1–5 μg/ml	*In vitro Pseudomonas aeruginosa* biofilms. MBEC used for antimicrobial assay.	MexAB-OprM efflux pump		No	Brooun et al., 2000
Ciprofloxacin	0.001–100 μg/ml					
Ofloxacin	0.1–100 μg/ml					
Erythromycin	6 μg/L	Biofilms of *P. aeruginosa* isolates, including multi-drug resistant strains recovered from urban sewage plants.	sul1, mexD (efflux pump), pilH (viruelnce gene)	(rhlR, lasI) quorum sensing genes	No	Bruchmann et al., 2013
Roxithromycin	1 μg/L					
Sulfamethoxazole	2 μg/L					
Kanamycin	0.8, 25.6 mg/L	*P. aeruginosa* and *P. protegens*, bacterial isolates grown in agar colonies			No	Chow et al., 2015
Tetracycline	25.6 mg/L					
Ciprofloxacin	0.0125, 0.025 mg/L					
Sulfadiazine	0.74–1.63 ng/g	ARGs occurrence and Abundance in natural biofilms in comparison to sediment, water column in estuarines influenced by WWTPs.	sul1, sul2, sul3, sulA, tetA, tetB, tetC, tetG, tetL, tetM, tetO, tetQ, tetS, tetT, tetW, tetX, ermB, Chl, qnrS, qnrB, aac(6′)-Ib, zntA, zntB	intI1	Yes	Guo et al., 2018
Sulfapyridine	0.44–11.46 ng/g					
Sulfathiazole	0.44–12.04 ng/g					
Sulfamethazine	1.24 ng/g					
Norfloxacin	16.26–129 ng/g					
Ciprofloxacin	11.73–66.27 ng/g					
Enrofloxacin	6.42–25.07 ng/g					
Ofloxacin	2.23–45.01 ng/g					
Tetracycline	1.51–7.80 ng/g					
Oxytetracycline	0.37–54.40 ng/g					
Doxycyclinehyclate	1.68–8.78 ng/g					
Chorotetracycline	0.85–7.76 ng/g					
Chloramphenicol	1.20–27.27 ng/g					
Eythromycin	1.33–4.68 ng/g					
Roxithromycin	0.41–3.35 ng/g					
Gentamicin streptomycin amikacin tobramycin	0.3 μg/ml	*P. aeruginosa* and *E. coli in vitro* biofilm formation assays.	arr (aminoglycoside response regulator)		Yes (2 spp)	Hoffman et al., 2005
Methicillin ampicillin amoxicillin cloxacillin	1 to 3 μg/ml	*In vitro* biofilms of 15 strains of methicillin-resistant *Staphylococcus aureus (MRSA).*		agr (quorum sensing system)	No	Kaplan et al., 2012
Triclosan	10 μg/L	River biofilms grown in bioreactors, algae and cyanobacteria effects were also tested.			Yes	Lawrence et al., 2009
Triclocarban	10 μg/L					
Sewage effluent	2.5 ppm	Addition of sewage effluent to river biofilms		intI1	Yes	Lehmann et al., 2016
Ciprofloxacin	461–1922 ng/L	*Staphylococcus spp.* and *Enterococcus spp.* from WWTP influent and effluent.			Yes (2spp)	Lépesová et al., 2018
Tetracycline	< 2–16 ng/L					
Erythromycin	22–102 ng/L					
Penicillin	< 1.3– < 2 ng/L					
Erythromycin	1 μg/L	River biofilms grown in artificial streams were exposed to a mixture of antibiotics and a combination of nutrient low to high concentrations	sul1, sul2	intI1	Yes	Subirats et al., 2018
Sulfamethozaxole						
Ciprofloxacin						
Erythromycin	1–4 μg/L	River biofilms grown in bioreactors. Biofilm thickness, bacterial biomass, and EPS volume against AB exposure was tested.			Yes	Waiser et al., 2016
Trimethoprim	4 μg/L					
Clindamycin	4 μg/L					
Kanamycin	25–100 μg/ml	*P. chloraphis in vitro* assays			No	Wang et al., 2016
Piperacillin	25–100 μg/ml					
Tobramycin	0.05, 0.1 and 0.3 mg/L	*Geobacter sulfurreducens* biofilms	acrA, acrB, ccpA, omcB, omcC, omcS, omcT, omcE, ppcA and ppcD	pilA-C, pilA-N, pilS, and pilC (pilus genes)	No	Zhou et al., 2017

*AB, Antibiotic; ARGs, Antibiotic Resistant Genes; MGEs, Mobile Genetic Elements; WWTP, Wastewater Treatment Plant; MBEC, minimum biofilm eradication concentration, EPS, Extracellular Polymeric Substance.*

### Structural Mechanisms of Resistance in Biofilms

In addition to genetic mechanisms of resistance, high heterogeneity and low permeability of biofilms allow for tolerance mechanisms which include persister cell development, altered cell permeability, and efflux pump regulation (Hall and Mah, 2017; Schroeder et al., 2017). The biofilm matrix and EPS do provide protection against exposure to a sustained stress by sub-MICs of antibiotics, and can offer an adaptive advantage to bacteria within biofilms by limiting the penetration of chemical stressors into deeper regions of the biofilm (Szomolay et al., 2005; Mangalappalli-Illathu et al., 2008b). Bacterial biofilms have also been shown to contain intrinsically resistant persister cells at a level of 1% of the total population that exhibit multi-drug and bactericidal agent tolerance (Lewis, 2005; Gebreyohannes et al., 2019). These dormant or slow-growing cells are generally identical to the rest of the population but display several phenotypic differences, such as resistance to normally lethal concentrations of antibiotics (Stewart and Costerton, 2001; Gebreyohannes et al., 2019; Jackson et al., 2019; Orazi and O’Toole, 2019). In contrast to antibiotic-resistant cells, persister cells exhibit temporary antibiotic-resistant phenotypes which have been found to last as long as 4 weeks (Miyaue et al., 2018).

Recently, two alternate HGT mechanisms facilitated by the biofilm structure were proposed (Abe et al., 2020); membrane vesicles and nanotubes, which are suggested to enable translocation of various cellular materials (possibly including ARGs) between adjacent biofilm bacteria. Extracellular membrane vesicles consist of 20 to 400 nm lipid-bilayer capsules produced and released by both Gram-positive and Gram-negative bacteria. They are ubiquitous in aquatic environments and have been reported in biofilms of *P. aeruginosa* (Murphy et al., 2014), *Escherichia coli* (Nakao et al., 2018) and *Vibrio cholerae* (Altindis et al., 2014). These outer membrane-derived vesicles have the potential to transport DNA, as well as RNA, proteins, metabolites and QS molecules (Abe et al., 2020). Nanotubes are membranous extracellular structures that can elongate to allow direct cell-to-cell contact. Unlike pili used in conjugation, nanotubes can transfer cytoplasmic material such as nutrients and DNA, thus they also have the potential to transfer ARGs to other bacteria. However, there is no current, direct evidence that demonstrates the transfer of ARGs through these two mechanisms in natural biofilms (Abe et al., 2020).

Biofilm cells also engage in QS, a process that enables bacteria to communicate *via* secreted signaling molecules called autoinducers. This process enables a population of bacteria to regulate gene expression collectively, and therefore control behavior on a community-wide scale. QS autoinducer signaling has also been associated with conjugation, phage induction and transformation (Abe et al., 2020). Antibiotics can act as signals for QS attenuators and there is evidence that QS controls the secretion of virulence factors, and formation of biofilms and conjugation, among other activities (Miller and Bassler, 2001). Vasudevan et al. (2018) describe more than 20 QS inhibitors (i.e., furanone, baicalein, lactonase, etc.) and their synergetic actions with antibiotics. In the absence of QS inhibitors, the biofilm develops a barrier and antibiotics are less able to enter the cell which leads to drug tolerance (Orazi and O’Toole, 2019).

## Antibiotic Resistance in Biofilms

Most studies analyzing the prevalence of ARGs in hotspots of antimicrobial resistance focus on a single species or clinically relevant strains tested *in vitro* (Høiby et al., 2010; Partridge et al., 2018). While the information extracted from these *in vitro* experiments is relevant, its noteworthy to remember environmental biofilms are typically formed by myriad species, and the effect after exposure to antibiotic residues or antibiotic-resistant bacteria would likely differ significantly from that of mono-species biofilms. Interactions between the biofilm community and other organisms can range from the biofilm acting as a barrier to allochthonous, incoming organisms, to biofilms serving as a source of diversity which expands membership and adds heterogeneity to key microbial components of the aquatic food chain (Balcázar et al., 2015).

Resistance assays also often simply demonstrate growth in the presence (or absence) of an antibiotic using traditional approaches (e.g., MIC). The ability of sub-MIC levels to act selectively on a population of organisms (i.e., biofilm) is currently not well understood in the context of innate vs. acquired resistance. Innate resistance refers to wildtype genes encoding antibiotic resistance or innate properties of the cell, whereas acquired resistance is conferred following chromosomal mutations or the acquisition of resistance genes through HGT (Fernández and Hancock, 2012; Hall and Mah, 2017; Schroeder et al., 2017). Both mechanisms are known features of biofilm communities, and as such contribute toward the development, maintenance and transmission of ARGs and general resistance (Hall and Mah, 2017). It is clear that more community-based exposure experiments conducted under controlled conditions are needed to provide meaningful data to broaden our understanding of how complex communities respond to stresses induced by very low concentrations of antibiotics.

### Biofilm Stressors and Antimicrobial Agents

It is widely accepted that bacteria living in biofilms are more resistant to chemical, physical and mechanical stresses than their planktonic counterparts (Li et al., 2001; Szomolay et al., 2005). The EPS matrix provides protection for the community from chemicals such as antibiotics, antivirals, pharmaceuticals, personal care products, disinfectants, heavy metals, pesticides and drugs (Mangalappalli-Illathu et al., 2008a; Høiby et al., 2010; Balcázar et al., 2015; Guo et al., 2018; Abe et al., 2020). Furthermore, genetic and physiological tolerance mechanisms induced by co-selection of metal and antibiotic resistance factors has been attributed to the sequestration properties of the biofilm matrix and the role of persister cells (Baker-Austin et al., 2006). Indeed, analysis of the occurrence and abundance of ARGs in naturally occurring biofilms in comparison to ARGs found in estuarine sediment and water exposed to antibiotics (sulfonamides, fluoroquinolones, tetracyclines, macrolides, and chloramphenicols) has confirmed that biofilms act as sinks for both types of contaminants (Guo et al., 2018).

Antibiotics in natural environments, including river biofilms, generally migrate from high concentration source reservoirs in the aqueous phase (and decrease with distance from those sources), enter wastewater systems, or directly impact pristine environments (Balcázar et al., 2015; Lehmann et al., 2016; Aubertheau et al., 2017; Grenni et al., 2018). The constant exposure of downstream microbial communities to low concentrations of antibiotics may influence the structure of microbial biofilm communities, and impose selection pressure for antimicrobial resistance, along with other collateral effects on ecosystem diversity (Quinlan et al., 2011; Kraemer et al., 2019; White and Hughes, 2019). Indeed, previous studies have found the total antibiotic concentration in biofilms was 2.3–5.8 times higher (51.7 ng/g–362.4 ng/g) than for sediment samples, with the abundance of ARGs such as *int*I1, *sul*1, *sul*2, *aac(6′)*-Ib, *tet*A, *tet*W was highest in biofilm samples, followed by sediment and water, respectively, (Guo et al., 2018).

It should be noted that recent studies suggest that the dispersal of ARGs or ARB from hotspots, including animal farms, are far more significant contributors to the environmental dissemination of ARGs than is *in situ* natural environmental selection (Brandt et al., 2015; Karkman et al., 2018; Zhao et al., 2020). Nevertheless, there is currently no consensus on the risk associated with sub-inhibitory concentrations of antibiotics regarding their abilities to select for antibiotic resistance and increase rates of bacterial evolution (Gillings and Stokes, 2012; Karkman et al., 2018; Zhao et al., 2020). Given the frequent domestic use of antibiotics, river biofilm communities are continuously exposed to these stressors. Understanding the ecological impact of long-term exposure of sub-inhibitory concentrations on a natural biofilm requires experimental knowledge of antibiotic concentration, exposure time, biofilm’s spatial distribution, biochemistry of the sorption sites, and the impact on biofilm structure, species abundance, and viability (Balcázar et al., 2015).

### Exposure to Sub-Inhibitory Antibiotic Concentrations

Although there is no single assay that is universally used to assess antimicrobial susceptibility in biofilms, plate-based phenotypic assays offer convenient tools for antimicrobial testing [e.g., the minimum biofilm eradication concentration (MBEC) assay]. Biofilm variants of this approach can also be used to determine the minimum inhibitory concentration (MIC) or the minimum biocidal concentration (MBC) (Hall and Mah, 2017; Thieme et al., 2019). The MIC refers to the lowest drug concentration that inhibits visible growth of target bacterial populations, and it is typically measured in planktonic mono-species cultures or on semi-solid agar surfaces (i.e., Kirby-Bauer disc diffusion). Antibiotic concentrations below the MIC are referred to as sub-inhibitory concentrations (sub-MICs), or minimal selective concentrations (MSC) (Gullberg et al., 2011). In surface waters worldwide, concentrations of antibiotics from treated sewage effluents and animal wastes tend to occur in the ng/L to mg/L range (Waiser et al., 2016). Although mg/L concentrations are considered very high, most aquatic systems have antibiotics present at ng/L concentrations (Chow et al., 2021). Furthermore, many antibiotics are excreted from animals and humans in a chemically active and environmentally persistent form, being detected downstream of wastewater treatment plants and adjacent to fields receiving animal manures (Finley et al., 2013).

It is estimated that the MSC for a diverse range of microorganisms ranges between 1/4 and 1/230 of the MIC values (Gullberg et al., 2011). It is also known that antibiotic concentrations 10–100 times lower than MIC values can increase the relative abundance of resistant bacteria and select for their resistance by accelerating the rate of adaptive evolution under *in vitro* conditions (Gullberg et al., 2011; Friman et al., 2015; Lundström et al., 2016; Danner et al., 2019). For example, sub-MIC antibiotic exposure has been shown to increase genetic diversity in microbial populations *via* the action of the bacterial SOS response, resulting in an increased mutation rate throughout the genome and *via* direct mutagenic effects on the DNA (Allen et al., 2010; Kraemer et al., 2019). Sustained MSC exposure can also select for the most resistant species (Romero et al., 2019), thereby altering both structure and function of the biofilm. For instance, the antimicrobials triclosan and triclocarban at concentrations of 10 μg/L for an 8-week period were shown to be highly toxic to autotrophs (algae and cyanobacteria), shifting river biofilms from an autotrophic community to one dominated by heterotrophs (Lawrence et al., 2009).

In WWTP systems, comparisons between influent and effluent biofilms show higher prevalence of antibiotic-resistant bacteria in effluent biofilms (Lépesová et al., 2018). Influent biofilms contained only vancomycin-resistant enterococci, while in effluent biofilms, resistance to 17 antibiotics was observed, with most *Enterococci* showing multi-drug resistance (MDR). Sub-MIC antibiotic exposures also have demonstrated effects on functional capacity of aquatic biofilms; efforts to profile the responses in biofilms exposed to sub-MICs of antibiotics have been performed downstream of WWTP discharges (Table 1). One such study which profiled the response of *P. aeruginosa* biofilms (including MDR strains) to sub-MIC concentrations of erythromycin (6 μg/L), roxithromycin (1 μg/L), and sulfamethoxazole (2 μg/L), revealed changes in biofilm dynamics that included biomass formation, spatial structure and expression of specific genes in different *P. aeruginosa* isolates (Bruchmann et al., 2013). Increases in quorum sensing (QS)-regulating gene activities were also observed after sub-MIC antibiotic exposure, with macrolides (i.e., erythromycin, roxithromycin) specifically upregulating QS-genes (*rhlR*, *lasI*). In contrast, the presence of sulfamethoxazole caused upregulation of *sul1* and the efflux pump, *mex*D, confirming the involvement of efflux pumps in sulfonamide resistance (Bruchmann et al., 2013). In many other cases, exposure to low concentrations of antibiotics were associated with mobile genetic element-mediated dissemination of ARGs through HGT (Celli and Trieu-Cuot, 1998; Bruchmann et al., 2013; Finley et al., 2013).

Interestingly, inlet and outlet samples of WWTP systems have demonstrated reductions in the copy numbers of ARGs after water treatment processes (Auguet et al., 2017), with authors quantifying ARGs *sul1* and *sul2* as the most abundant in both biofilms and water columns, with roughly 1 resistance gene for each 10 copies of 16 s RNA gene. Moreover, significant differences were observed for *intI1* (gene encoding integrase of Class I integrons) between biofilms found at sewage outlet (1.60 log) and inlet (1.16 log) locations. Class 1 integrons are often physically linked to multiple resistance determinants for antibiotics, and thus used as a proxy for ARGs of anthropogenic origin (Zhao et al., 2020). These results thus confirm biofilms as a reservoir for mobile genetic elements. Constant exposure to antibiotics over a range of sub-MICs would thus be expected to encourage HGT (Kraemer et al., 2019) and ARB development.

Comparison between resistomes exposed to higher concentrations of antibiotics (e.g., WWTPs) and lower concentrations of antibiotics (e.g., receiving water bodies of agriculture run-off, or other down-gradient systems) require further study to corroborate the transfer and acquisition of antibiotic resistance under these conditions (Mao et al., 2015; Ebmeyer et al., 2021). Additionally, studies on the emergence of MDR and virulence factors should continue to be undertaken to better understand the risk and spread of these factors in environmental resistomes.

## The Role of Animal Agriculture in Environmental Antibiotic Resistance

In addition to the release of antibiotic residues *via* the discharge of effluent from WWTPs, agriculture waste products and associated field run-off following application of animal waste as source of fertilizer represents a second major contributor to environmental antibiotic resistance (Topp et al., 2018; Goulas et al., 2020; Henriot et al., 2021). The demand for animal protein has been increasing over the last decade and global trends indicate intensive animal production will continue to increase the occurrence of ARB (Tiseo et al., 2020; Wang et al., 2021). It is estimated that 73% of all antimicrobials sold globally are used to raise animals for human consumption (Van Boeckel et al., 2019). While many countries have legislated tighter controls over antibiotic use (Zhao et al., 2020), others continue to permit prophylactic antibiotic usage in animal operations (Finley et al., 2013; Kraemer et al., 2019).

It is abundantly clear that exposure of microorganisms to veterinary/agricultural antibiotic residues can contribute to the development of ARGs and ARB (Manaia et al., 2020; Wang et al., 2021); however, little is known about the selective pressure that sub-MIC concentrations of antibiotics play on initial microbial colonization processes as well as biofilm development in receiving environments along with the environmental resistome (Yang et al., 2020). The environmental resistome influenced by the agriculture sources is characterized by two processes: extended periods of selective pressure imposed by antibiotic residues, and the subsequent dispersion of ARGs or ARB (Zhao et al., 2020). Dispersion is enhanced by the incomplete biodegradation of antibiotics, as the half-life of antibiotics in manure are estimated to be between 2 and 100 days. Released antibiotics are chemically diverse and degrade at different rates in the environment *via* sorption, photodegradation oxidation, and biodegradation (Grenni et al., 2018), making it difficult to predict how quickly they degrade and the extent to which they might continue to exert selective pressure for ARB development (O’Neill, 2016; Manyi-Loh et al., 2018).

It is noteworthy that unimpacted environmental resistomes deemed to be “pristine” with respect to anthropogenic activities, are increasingly difficult to find (Hooban et al., 2020). Widespread environmental pollution from antibiotic production facilities or agricultural operations has necessitated the call to monitor wildlife, agriculture, aquaculture systems, along with supposedly unimpacted environmental sites (Khan et al., 2013; Wellington et al., 2013; Thakur and Gray, 2019). In agricultural systems, veterinary drugs reach the surrounding environments, often in a metabolically-active form, through distribution and disposal of livestock manure and urine (Zhao et al., 2020). Manure is a common fertilizer used in farmland soils and crop production, and due to its potential to contribute to ARG dissemination to soils, aquatic systems, including plant microbiomes, it is a practice of ongoing concern (Zhao et al., 2020).

With regard to agriculture and aquaculture practices, 51 antibiotics have been reported as being in use according to the World Health Organization (WHO, 2017), including the six most-common classes of antibiotics: tetracyclines, quinolones, aminoglycosides, polymyxins, macrolides, penicillins, and sulfonamides (Chekabab et al., 2021). It is important to note that antibiotic usage for humans and animals often occurs within similar drug classes (e.g., gentamicin/aminoglycoside, ampicillin/ β-lactams, ciprofloxacin/fluoroquinolones, trimethoprim/antifolate, and tetracycline) (Finley et al., 2013; EUCAST, 2019); thus, the potential for cross-resistance to those drugs used in either setting exists. For instance, in soil microbiomes exposed to manure (Han et al., 2018), 163 ARGs were identified in untreated soils whereas 245, 230, and 245 ARGs were detected in soils treated with manure from poultry, swine and cattle, respectively. Of the total ARGs, the highest abundance was found to encode β-lactamases (19.6%), multi-drug resistance (18.8%) and macrolides (15.4%).

Tetracyclines and macrolides have historically seen extensive use in agriculture (Ebmeyer et al., 2021) and ARGs conferring resistance to these antibiotics are among the most prevalent (Chekabab et al., 2020, 2021). In China, most of the veterinary antibiotics consist of tetracyclines and sulfonamides (Zhou et al., 2020). Manure, soil and water samples of sixteen animal farms in Southeastern China were sampled to determine the abundance of ARGs (Wang et al., 2016); 22 ARGs were found to confer resistance to five major classes of antibiotics including tetracyclines, sulfonamides, quinolones, aminoglycosides, and macrolides. The author’s results showed that the spread of the agriculture-impacted resistome was dominated by *sul* genes, which were the most extensive, followed by *tet* and *erm* genes.

Metals can also act as co-selective agents driving the enrichment of genetic elements which contain resistance genes to both metals and ARGs (Baker-Austin et al., 2006; Zhao et al., 2020). The co-selection of metals and ARGs has been recorded in agriculture-impacted resistomes (Zhao et al., 2020). In some agricultural settings, Cu, As, and Zn continues to be used as supplementary feed additives for animal growth promotion and disease prevention (Poole, 2017).

Opportunistic bacterial pathogens such as *Aeromonas* spp., *Citrobacter* spp., *Enterobacter* spp., *Mycobacterium* spp., *Legionella* spp., *Staphylococcus* spp., *Enterococcus* spp., *P. aeruginosa*, and *K. pneumoniae* naturally occur in aquatic and soil environments and can persist and grow in biofilms of drinking water systems (Schwartz et al., 2003; Obst et al., 2006; Wingender and Flemming, 2011; Ahmad et al., 2021). Agricultural practices, such as irrigation, as well as drinking water distribution systems in which biofilms are known to form, have also been suggested to act as reservoirs for pathogenic microorganisms (Wang et al., 2021). As biofilms develop, resistant bacteria can be released to continue their life cycle as planktonic cells, and subsequently colonize, or disperse to, other ecosystems carrying their ARG compliment. Additionally, pathogenic bacteria such as *P. aeruginosa* and *Legionella pneumophila* that are resistant to an antibiotic can survive sewage treatments and migrate downstream to eventually be included in a biofilm (Wingender and Flemming, 2011). Most bacterial populations inhabiting freshwater biofilms are non-pathogenic. However, non-pathogenic bacteria have a higher chance of interacting with pathogenic bacteria in high-density systems such as biofilms (Wingender and Flemming, 2011).

Veterinary antibiotic usage over the last several years have been reduced worldwide as result of greater awareness of the threat of antibiotic resistance (Kraemer et al., 2019); however, agricultural antibiotic use still remains a major source of environmental antibiotic pollution (Wang et al., 2021). Overall, the effects of sub-MIC antibiotics outside clinical settings have not yet been as fully explored nor monitored possibly due to detection difficulties pose by low effective concentrations. However, recent studies support the premise that acquisition of resistance can occur at sub-MIC antibiotic concentrations (Marti et al., 2014; Chow et al., 2015, 2021). Clearly, more experimental data is required to further comprehend the risk of exposed to low dosages of antimicrobials, as well as the response interactions from the entire microbial community and not just that of individual bacteria.

## Bacteriophage Dynamics in Biofilms and the Environmental Resistome

Over the past decade, studies of the ecology bacterial viruses (bacteriophage) have become more feasible thanks to the increased accessibility of high-throughput sequencing methods. However, a clear understanding of the ecological role of bacteriophage in aquatic environments, including freshwater biofilms, is still lacking (Battin et al., 2016; Bekliz et al., 2019; Palermo et al., 2019). This lack of knowledge of environmental phage is compounded by the inability to characterize the majority of bacterial viruses, with over 65% of viral sequences from surface waters being unrecognized when compared to existing databases (Palermo et al., 2019).

Flood and Ashbolt (2000) observed that wetland biofilms could entrap viral-sized particles and concentrate them over 100-fold, compared to suspended numbers in the surrounding water column. Further, in the different stages of WWTPs, phage have been observed at concentrations ranging from 10^8^ to 10^10^ ml^–1^, which is 10–1,000 times higher than in natural (unimpacted) aquatic environments, indicating WWTPs as important reservoirs of bacteriophage (Wu and Liu, 2009). While bacteriophage do not represent a direct threat to human or domestic animals, they shape the diversity and genetic architecture of their hosts (Chopyk et al., 2020). Since there are records of phage invading biofilms by disrupting the EPS matrix or by killing embedded cells (Sutherland et al., 2004), it was originally believed that biofilm formation and phage transmission were mutually exclusive (Abe et al., 2020). However, more recent work has described phage as playing supporting beneficial roles in high density microbial colonies such as biofilms, as cell lysis mediated by phage infection often leads to production of e-DNA. The e-DNA released by phage may then be integrated by the biofilm’s matrix, thus increasing the opportunity for acquisition of resistance genes through HGT (Fernández et al., 2018).

Most studies of bacteriophage composition have focused on WWTP microbial communities, where the order *Caudovirales* comprises most of the viral sequences, with dominant viral families including *Myoviridae*, *Siphoviridae*, and *Podoviridae* (Parsley et al., 2010; Allen et al., 2011; Cantalupo et al., 2011; Tamaki et al., 2012; Chopyk et al., 2020; Petrovich et al., 2020). A similar virome composition was observed in benthic biofilms of three different freshwater streams, where *Siphoviridae*, *Podoviridae* and *Myoviridae* were the dominant viral taxa (Bekliz et al., 2019). Moreover, it was observed that ARGs such as *sul1*, *bla*_*TEM*_ and *bla*_*CTX*–*M*_, were more persistent after WWTP treatments (chlorination, thermal treatment and natural inactivation) when more of these ARGs were found within the protein capsid of phage particles than found within bacteria (Calero-Cáceres and Muniesa, 2016). It is thought that the protein capsid could provide DNA fragments with enough protection to survive various environmental stressors, thereby serving as a significant reservoir. Furthermore, the possibility of ARGs being transferred to a suitable host would also increase under such a scenario (Calero-Cáceres and Muniesa, 2016).

One study that described both the bacterial and viral composition of the swine gut identified ARGs such as MDR efflux pumps [*bcrA*, *macB*, *mef(A)*] within the virome and that the abundance of phage integrase-encoding genes were significantly increased in the viromes of medicated swine relative to non-medicated swine, indicating induction of bacteriophage in the presence of antibiotics (Allen et al., 2011). However, the authors did not observe significant changes in bacteriophage abundance, suggesting that while they were induced in the swine gut, this did not result in the transfer of ARGs *via* prophage to bacteria in the swine gut. In contrast, a study by Zhang and LeJeune (2008) demonstrated that *bla*CMY-2, *tetA* and *tetB* were transferred *via* phage between *Salmonella* serovars of bovine origin, providing evidence of ARG propagation by phage-mediated transduction events.

Bacteriophage are becoming increasingly recognized for their potential role in the dissemination of antimicrobial resistance, as well as other genes that shape bacterial community composition (i.e., virulence factors, auxiliary metabolic genes) (Colavecchio et al., 2017; Breitbart et al., 2018; Chopyk et al., 2020). Evidence suggests that dissemination of ARGs by bacteriophage may be possible between different, and even remote, environments not contaminated by antibiotics as a result of anthropogenic activities (Muniesa et al., 2013). Just as exposure to antibiotics leads to selective pressure on susceptible bacteria, the constant presence of phage and/or competing viruses may exert a selective pressure on microbial communities, influencing the prevalence and abundance of antibiotic resistant genes (Madsen et al., 2012; Beceiro et al., 2013). In studies that compared viral and bacterial taxonomic composition, dsDNA phage were the most abundant of the viral members observed, though it was noted that virome studies targeting bacteriophage, and the availability of sequenced viromes, is currently limited (Calero-Cáceres et al., 2019).

There is some evidence to suggest viral taxa may not have a great impact on ARG transfer *via* transduction (Colavecchio et al., 2017; Enault et al., 2017). However, transduction tends to be more common in sites with high viral and bacterial density (e.g., WWTPs) (Petrovich et al., 2020), suggesting a different pattern for biofilms where their high microbial diversity and density could possibly enable them to be more susceptible to viral-ARG associations. Nonetheless, there is an ongoing debate on whether viruses are associated with antimicrobial resistance spread and if they contribute significantly to HGT (Parsley et al., 2010; Enault et al., 2017; Petrovich et al., 2020). Notably, very few studies have compared the dynamics of bacterial and viral communities in biofilms. Moreover, few studies have attempted to correlate the effects of antiviral drugs in combination with antibiotics on microbial communities, and monitoring of antivirals in wastewaters, surface waters, groundwater, and drinking water is not currently conducted. However, some antivirals are highly bioactive and are proposed to negatively affect non-target organisms and persist in aquatic environments (Jain et al., 2013; Sims and Kasprzyk-Hordern, 2020). For this reason, it is important to investigate the simultaneous occurrence of antiviral drugs with antibiotics.

Further research is clearly needed to understand associations between bacterial and viral taxa, particularly in critical environmental niches (e.g., biofilms) to better address whether viruses contribute to the spread or transfer of ARG. More insight into the relations of bacteriophage and the prevalence of antimicrobial resistance would provide data for improved methods for effluent water treatment, better comprehension of the interactions between viromes and sub-MIC-exposed environmental resistomes, and offer a new framework for monitoring strategies to mitigate the spread of pathogenic viruses and antimicrobial resistance.

## Conclusion

While many studies of ARG and ARB spread have focused on hotspots where high antibiotic concentrations are released (e.g., hospitals, WWTPs and large animal production facilities), the majority of downstream environments are routinely exposed to much lower concentrations of these metabolically-active agents. The extent to which these more prevalent antibiotic contaminants drive proliferation of antibiotic resistance in the environmental resistome remains to be fully elucidated. Interactions of antiviral and antibiotic drugs between the microbial community of pristine (unimpacted) environmental reservoirs are complex and also poorly understood, although seemingly offer myriad non-mutually exclusive events with potential to impact the persistence and spread of antibiotic resistance.

Most environmental resistomes are becoming increasingly influenced by the constant selective pressure from human activities, and in particular, from industrialized agriculture practices. In this review, we have focused on the effects of low antibiotic concentration exposure of biofilms colonizing freshwater environments and as a result of agricultural practices given the growing evidence suggesting sub-MIC selectively influences the prevalence of ARGs and ARB. Furthermore, the rich microbial diversity of biofilms commonly includes high concentrations of viruses, and in particular, bacteriophage, which are a potentially important and under-appreciated mechanism of antibiotic resistance propagation. The environmental virome as an integrated part of the environmental resistome, and specifically the bacteriophage’s role in the environmental transfer of antimicrobial resistance, requires more study to better understand the role of the environment in antibiotic resistance transmission.

## Author Contributions

GF-V: writing—original draft. JB: supervision and writing—review and editing. JRL: writing—review and editing. DRK: supervision, writing—review and editing, and funding acquisition. All authors contributed to the article and approved the submitted version.

## Conflict of Interest

The authors declare that the research was conducted in the absence of any commercial or financial relationships that could be construed as a potential conflict of interest.

## Publisher’s Note

All claims expressed in this article are solely those of the authors and do not necessarily represent those of their affiliated organizations, or those of the publisher, the editors and the reviewers. Any product that may be evaluated in this article, or claim that may be made by its manufacturer, is not guaranteed or endorsed by the publisher.
